# Impact of Human Cytomegalovirus and Human Herpesvirus 6 Infection on the Expression of Factors Associated with Cell Fibrosis and Apoptosis: Clues for Implication in Systemic Sclerosis Development

**DOI:** 10.3390/ijms21176397

**Published:** 2020-09-03

**Authors:** Maria-Cristina Arcangeletti, Maria D’Accolti, Clara Maccari, Irene Soffritti, Flora De Conto, Carlo Chezzi, Adriana Calderaro, Clodoveo Ferri, Elisabetta Caselli

**Affiliations:** 1 Department of Medicine and Surgery, Unit of Virology, University-Hospital of Parma, University of Parma, 43126 Parma, Italy; clara.maccari@unipr.it (C.M.); flora.deconto@unipr.it (F.D.C.); carlo.chezzi@unipr.it (C.C.); adriana.calderaro@unipr.it (A.C.); 2 Department of Chemical and Pharmaceutical Sciences, Section of Microbiology and Medical Genetics, University of Ferrara, 44121 Ferrara, Italy; maria.daccolti@unife.it (M.D.); irene.soffritti@unife.it (I.S.); elisabetta.caselli@unife.it (E.C.); 3 Department of Medical and Surgical Sciences for Children and Adults, Rheumatology Unit, University-Hospital Policlinico of Modena, University of Modena and Reggio Emilia, 41121 Modena, Italy; clodoveo.ferri@unimore.it

**Keywords:** systemic sclerosis, fibrosis, apoptosis, human cytomegalovirus, human herpesvirus 6

## Abstract

Systemic sclerosis (SSc) is a severe autoimmune disorder characterized by vasculopathy and multi-organ fibrosis; its etiology and pathogenesis are still largely unknown. Herpesvirus infections, particularly by human cytomegalovirus (HCMV) and human herpesvirus 6 (HHV-6), have been suggested among triggers of the disease based on virological and immunological observations. However, the direct impact of HCMV and/or HHV-6 infection on cell fibrosis and apoptosis at the cell microenvironment level has not yet been clarified. Thus, this study aimed to investigate the effects of HCMV and HHV-6 infection on the induction of pro-fibrosis or pro-apoptosis conditions in primary human dermal fibroblasts, one of the relevant SSc target cells. The analysis, performed by microarray in in vitro HCMV- or HHV-6-infected vs. uninfected cells, using specific panels for the detection of the main cellular factors associated with fibrosis or apoptosis, showed that both viruses significantly modified the expression of at least 30 pro-fibrotic and 20 pro-apoptotic factors. Notably, several recognized pro-fibrotic factors were highly induced, and most of them were reported to be involved in vivo in the multifactorial and multistep pathogenic process of SSc, thus suggesting a potential role of both HCMV and HHV-6.

## 1. Introduction

Systemic sclerosis (SSc) is a chronic autoimmune disease characterized by immunological abnormalities, vasculopathy, and excessive extracellular matrix deposition, which leads to vascular involvement, apoptosis, and fibrosis of the skin and internal organs [1,2,3,4,5,6,7].

Although SSc patients can present extremely heterogeneous clinical pictures, two well-recognized subgroups have been identified according to the extent of skin involvement: patients with widespread skin involvement (diffuse cutaneous subset; dcSSc) and patients with limited skin involvement (limited cutaneous subset; lcSSc) [8]. DcSSc patients develop very quickly fibrosis of the skin, lungs, and other internal organs (e.g., heart, gastrointestinal tract, kidneys, tendons, and ligaments); specifically, dcSSc is responsible for a higher mortality rate, although the course of the disease is extremely variable. On the contrary, lcSSc patients mainly present vascular abnormalities and the disease often has a more favorable outcome.

The pathogenesis of SSc remains largely unknown, however accumulating evidence suggests that the disease could be the result of a multistep and multifactorial process. Among the involved/predisposing factors, several genes have been associated with distinct SSc phenotypes and a positive family history represents a strong risk factor [9,10]. Additionally, oxidative stress has been evoked as an important element in the pathogenesis of SSc [11,12], as well as environmental factors [13,14]. Moreover, persistent/latent viral infections, such as human cytomegalovirus (HCMV) and human herpesvirus 6 (HHV-6) infections, have been evoked as possibly involved in the pathogenesis of SSc [13,15,16]. Both viruses belong to the *Betaherpesvirinae* subfamily, are genetically related, and have a worldwide distribution, sustaining primary infection usually early in life and then establishing a latent infection lifelong in the host.

A possible role of HCMV in the etiopathogenesis of SSc has been postulated on the basis of several lines of evidence [17,18,19,20,21,22,23]. First of all, HCMV is able to infect in vivo the “hallmark” cells of SSc, represented by fibroblasts and endothelial cells [24,25]. Another piece of evidence supporting HCMV involvement in the pathogenesis of SSc is the detection of viral transcripts in endothelial cells from skin biopsy of a woman with SSc diagnosed after an acute HCMV infection [26].

Moreover, with respect to humoral immunity, significantly higher levels of anti-HCMV antibodies were detected in SSc patients compared to the healthy subjects, in particular directed against the immunodominant viral antigens, such as the tegument phosphoprotein pp65, the major immediate-early protein IE1 and the product of the viral UL94 gene [22,27,28,29,30].

It has also been hypothesized, evoking a mechanism of molecular mimicry, a role of antibodies directed against HCMV UL94 gene product (among those most frequently detected in the serum of SSc patients) in the recognition of membrane receptors of fibroblasts and endothelial cells with subsequent expression of genes functionally associated with clinical signs of SSc [27,31,32].

As regards cell-mediated immunity, a relevant role of T lymphocyte responses and pro-inflammatory cytokine aberrant production in the pathogenesis of SSc has been highlighted, with their possible contribution in the modulation of fibrosis and vascular damage [17,33,34,35,36,37,38,39,40]. Recent data from our group support the importance of HCMV specific CD8+ T cells, demonstrating a statistically significant association of HCMV-antigen driven CD8+ T cell responses in SSc patients with some of the most relevant disease parameters [16].

On the other hand, HHV-6 infection has been repeatedly reported as a possible triggering agent in SSc development, although there is still little information about the possible mechanisms underlying its role in the disease [41]. Two species are recognized, HHV-6A and 6B, showing high genome homology but dissimilar tropism and pathogenic associations [42]. Similarly to HCMV, HHV-6 has a tropism for endothelial cells [43], where it was found in an active replicative state in vivo [43], and can infect endothelial cells of different origin in vitro, inducing secretion of pro-inflammatory cytokines [44]. Of note, HHV-6 infection can impair the pro-angiogenetic ability of vascular and lymphatic endothelial cells, thanks to the action of the virus U94 gene product [45]. This is of particular note, since endothelial injury is one of the first steps in the pathogenesis of SSc, mostly affecting microcirculation [46]. Besides, HHV-6 was detected with high frequency in thyroid cells of subjects affected by Hashimoto’s autoimmune thyroiditis, a condition often preceding SSc disease [47], and its infection/reactivation has been associated to several autoimmune pathologies, including multiple sclerosis, Sjogren syndrome, rheumatoid arthritis, systemic lupus erythematosus, Purpura fulminans, severe autoimmune acquired protein S deficiency, and severe autoimmune hepatitis [48,49,50,51,52,53,54,55]. More recently, HHV-6 was also detected with high frequency in the blood and skin tissue of SSc patients [15], who also exhibited an anti-U94 antibody titer significantly higher than controls, suggesting that SSc subjects may undergo multiple virus reactivations. Notably, HHV-6A, and not HHV-6B, was detected at the tissue level, confirming the different tropism and pathogenic action of the two species. Furthermore, HHV-6A was shown to induce the expression of pro-fibrosis factors in infected vascular endothelial cells [15], suggesting its possible role in endothelial injury during SSc.

Based on these observations, this study aimed to assess the impact of HCMV and HHV-6A infection on the expression of pro-fibrotic and pro-apoptotic factors in primary human dermal fibroblasts, since they are among the specific target cells of SSc.

## 2. Results

### 2.1. HCMV and HHV-6A DNA Quantitative Evaluation in Primary Human Dermal Fibroblasts at Different Times Post-Infection In Vitro

Primary human dermal fibroblasts were infected with HCMV TB40E strain at a MOI of 0.1. At 0, 4, 7, 10, and 14 days post-infection (p.i.) cells were harvested and processed for DNA extraction and the efficiency of HCMV infection was evaluated by q-Real-Time PCR targeting HCMV immediate-early (IE)1 gene at the aforementioned times. The results show that HCMV DNA copies/mL gradually increased from day 4 to day 14 p.i. in parallel with the characteristic cytopathic effect (Table 1).

Human dermal fibroblasts were in parallel infected with HHV-6A strain U1102 at a MOI of 1. Samples of infected cells were collected at the same times p.i. described for HCMV (namely 0, 4, 7, 10, and 14 days p.i.) and total DNA was extracted. Efficiency of HHV-6A infection was evaluated by a specific qPCR targeting U94 virus gene. The results showed, as expected, an initial increase of HHV-6A DNA (from day 4 to 7 p.i.) followed by a gradual decrease till the end of the experiment (14 days p.i.), suggesting the initial establishment of an active replication rapidly followed by a latent infection (Table 2). No cytopathic effect was observed as a result of virus infection.

### 2.2. Induction of Fibrosis-Associated Transcripts in HCMV- or HHV-6A-Infected Primary Human Dermal Fibroblasts

**Figure 1:**
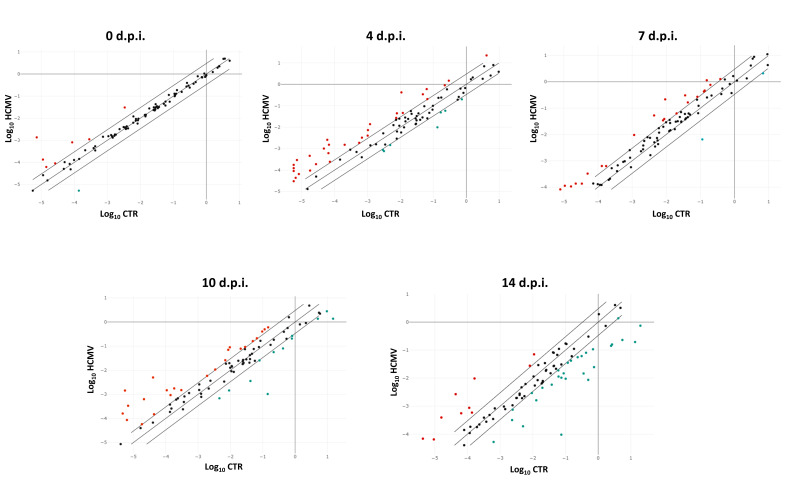
Scatterplot representation of the fibrosis-associated factors altered by human cytomegalovirus (HCMV) infection in primary human dermal fibroblasts. At each time p.i. (d.p.i. = days post-infection), cell samples were collected and analyzed by specific qPCR microarray. The significance threshold was put at 3-fold expression change in infected vs. uninfected control cells. Red dots: upregulated factors; blue dots: downregulated factors; black dots: not significantly altered factors. Results represent mean values of duplicate samples from two independent experiments, and are expressed in a logarithmic scale (Log_10_ HCMV = logarithmic values in HCMV infected cells; Log_10_ CTR = logarithmic values in control uninfected cells).

Analysis of the expression of pro-fibrosis transcripts, performed by qPCR microarray on RNA extracted from HCMV infected cells, shows that several factors involved in the development of fibrosis are up- or downregulated in infected fibroblasts (Figure 1).

In particular, already after the adsorption period (day 0 p.i.), four factors out of the 84 analyzed resulted to be significantly altered in HCMV-infected fibroblasts compared to uninfected cells. At this time point, a strong expression of the pro-inflammatory cytokine Tumor Necrosis Factor-α (TNF-α; 186.37 fold compared to uninfected cells) and a significant upregulation of Plasminogen Activator Inhibitor-1 (PAI-1 or SERPINA1; 11.99 fold), Chemokine (C-C motif) ligand 2 (CCL2; 9.08 fold), and Interleukin 1 beta (IL-1β or IL-1B; 9.36 fold) was observed.

Other induced factors, although to a lesser extent, included Chemokine (C-C motif) ligand 11 (CCL11; 3.9 fold), Matrix Metalloproteinase-9 (MMP9; 4.39 fold), and Bone Morphogenic Protein 7 (BMP7; 3.44 fold). At day 4 p.i., 15 transcripts associated with fibrosis were highly upregulated. In detail, CCL11 (43.1 fold), CCL2 (39.13 fold), IL-1β (41.08 fold), TNFα (30.6 fold), and Plasminogen (PLG; 26.29 fold) transcripts resulted strongly upregulated, followed by SERPINA1 (22.53 fold), Interleukin 13 (IL-13; 21.39 fold), Matrix Metalloproteinase-3 and -13 (MMP3, 5.8 fold; MMP13, 21.51 fold), Chemokine Receptor type 4 (CXCR-4; 15.82 fold), Hepatocyte Growth Factor (HGF; 11.59 fold), Fas ligand (FASLG or CD95L; 9.48 fold), Matrix Metalloproteinase-1 (MMP1; 9.51 fold), Chemokine (C-C motif) ligand 3 (CCL3; 7.96 fold), and BMP7 (6.65 fold). In the following days p.i., CCL2 transcript continued to be significantly upregulated at days 7 and 10 p.i. (23.1 and 9.29 fold, respectively); BMP7 and CXCR-4 resulted the most upregulated factors at day 10 p.i. (261.31 and 123.08 fold, respectively) with CCL3 (48.74 fold), TNFα (34.21 fold), SERPINA1 (13.67 fold), and IL-13 (9.61 fold). At day 14 p.i., a significantly high expression was observed for BMP7 (61.34 fold), CXCR-4 (59.15 fold), MMP13 (15.94 fold), SERPINA1 (7.69 fold), and TNFα (6.92 fold). Finally, the expression of IL-13 receptor subunit alpha 2 (IL13RA2) resulted gradually downregulated at day 4 p.i. (13.42 fold), at day 7 p.i. (17.57 fold), and at day 10 p.i. (138.44 fold), with a strong decrease observed at day 14 p.i. (776.73 fold).

**Figure 2:**
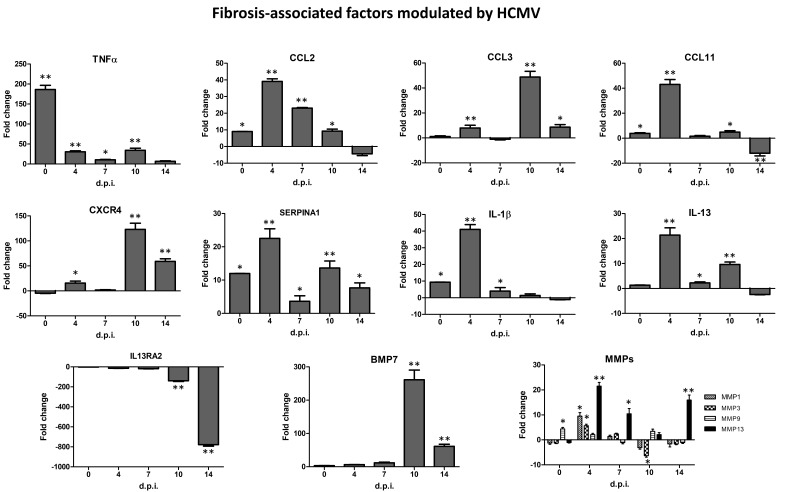
Expression kinetics of fibrosis-associated factors most significantly modulated by human cytomegalovirus (HCMV) infection in primary human dermal fibroblasts. At each time p.i. (d.p.i. = days post-infection), cell samples were collected and analyzed by specific qPCR microarray. Results are expressed as mean values of fold change (infected cells vs. controls) ± SD of duplicate samples from two independent experiments. * *pc* < 0.01; ** *pc* < 0.001.

The expression kinetics of the most HCMV-induced fibrosis-associated factors are shown in Figure 2.

**Figure 3:**
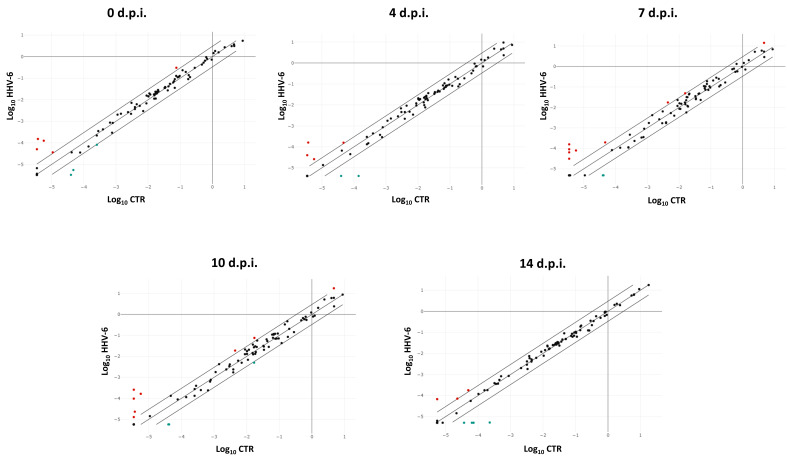
Scatterplot representation of the fibrosis-associated factors altered by human herpesvirus 6A (HHV-6A) infection in primary human dermal fibroblasts. At each time p.i. (d.p.i. = days post-infection), cell samples were collected and analyzed by specific qPCR microarray. The significance threshold was put at 3-fold expression change in infected vs. uninfected control cells. Red dots: upregulated factors; blue dots: downregulated factors; black dots: not significantly altered factors. Results represent mean values of duplicate samples from two independent experiments, and are expressed in logarithmic scale (Log_10_ HHV-6 = logarithmic values in HHV-6A-infected cells; Log_10_ CTR = logarithmic values in control uninfected cells).

Similar to what was detected in HCMV infected cells, the analysis of pro-fibrosis factors expression in HHV-6A infected cells showed a significant modulation by virus infection compared to uninfected control cells, although the number of modulated factors and the extent of the altered expression was lower compared to what was observed with HCMV (Figure 3).

Immediately after virus adsorption to fibroblasts, some factors appeared modulated, although to a limited extent compared to HCMV. In detail, virus binding/entry induced the upregulation of CXCR4 (4.66 fold), IL-1β (3.21 fold), and the matrix metalloproteinases MMP1, 3, and 9 (respectively, 4.63, 6.32, and 4.63 fold), whereas IL-1α was downregulated (−12.75 fold). At the subsequent times, the upregulated expression of such genes was confirmed and even increased. CXCR4 was hyper-expressed at all times p.i. (36.81 fold at 4 days p.i., 10.1 at 7 days p.i., 5.29 at 10 days p.i., and 3.48 at 14 days p.i.); IL-1β was upmodulated at all times p.i. as well (5.41, 3.27, 6.84, and 7.66 fold at 4, 7, 10, and 14 days p.i.) and MMPs were similarly maintained upregulated (MMP1 9.47 fold, MMP13 17.3 fold, MMP3 21.55 fold, MMP9 6.14 fold). In addition, the expression of IL-10 was constantly upregulated from 4 to 14 days p.i. (up to 20.7 fold), that of IL-4 (up to 51.02 fold). Instead, IL-1α was downmodulated at all times p.i. (−64.75, −33.28, −31.37, and −33.98 fold at 4, 7, 10, and 14 days p.i.). Additionally, the Integrin αvβ6 (ITGB6) expression increased at 4, 7, and 14 days p.i. (9.12, 4.15, and 18 fold, respectively), and a significant upregulation of TNFα was detected at 7 and 10 days p.i. (28.0 and 17.10 fold, respectively).

**Figure 4:**
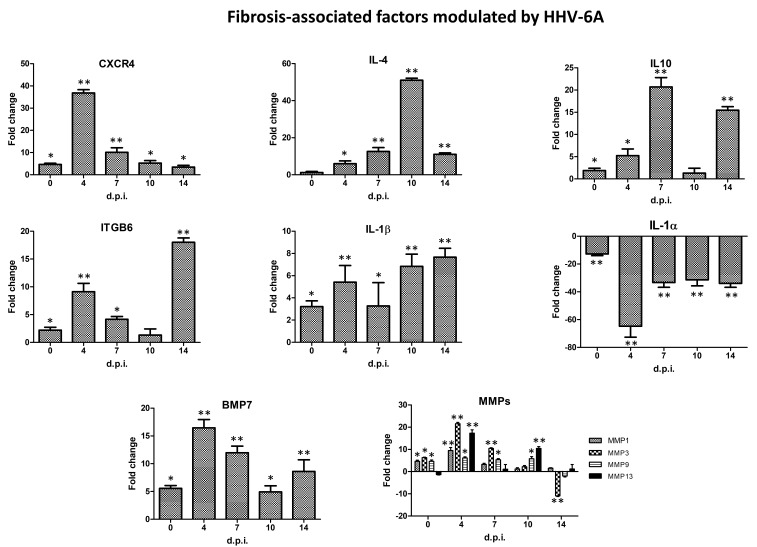
Expression kinetics of fibrosis-associated factors modulated by human herpesvirus 6A (HHV-6A) infection in primary human dermal fibroblasts. At each time p.i. (d.p.i. = days post-infection), cell samples were collected and analyzed by specific qPCR microarray. Results are expressed as mean values of fold change (infected cells vs. controls) ± SD of duplicate samples from two independent experiments. * *pc* < 0.01; ** *pc* < 0.001.

Similar to HCMV, also HHV-6A induced the expression of BMP7 very early, starting just after adsorption (5.56 fold), and at the following times p.i. (16.46, 11.97, 4.93, and 8.61 fold at 4, 7, 10, and 14 days p.i., respectively). Figure 4 shows the expression kinetics of the most HHV-6A induced fibrosis-associated factors.

Of note, among the most induced factors, six were up- or downmodulated by both HCMV and HHV-6A, namely CXCR4, IL-1β, MMP1, MMP13, TNFα, and BMP7.

### 2.3. Altered Expression of Apoptosis-Associated Transcripts in HCMV- or HHV-6A-Infected Primary Human Dermal Fibroblasts

**Figure 5:**
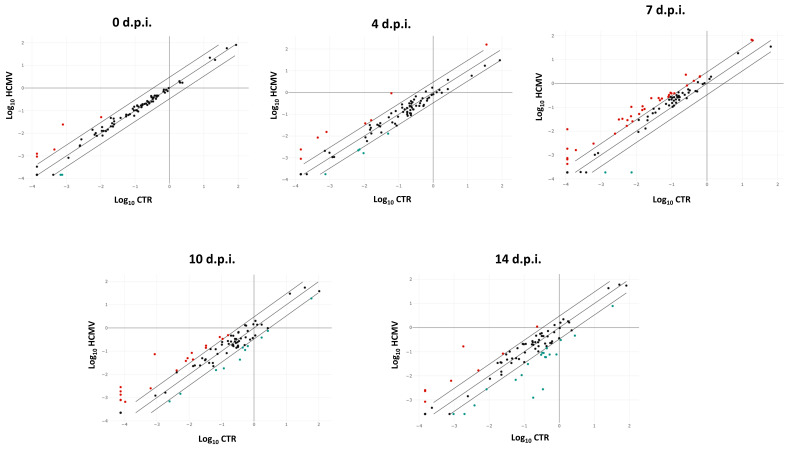
Scatterplot representation of the apoptosis-associated factors altered by human cytomegalovirus (HCMV) infection in primary human dermal fibroblasts. At each time p.i. (d.p.i. = days post-infection), cell samples were collected and analyzed by specific qPCR microarray. The significance threshold was put at 3-fold expression change in infected vs. uninfected control cells. Red dots: upregulated factors; blue dots: downregulated factors; black dots: not significantly altered factors. Results represent mean values of duplicate samples from two independent experiments, and are expressed in logarithmic scale (Log_10_ HCMV = logarithmic values in HCMV infected cells; Log_10_ CTR = logarithmic values in control uninfected cells).

A number of apoptosis-associated genes were identified by qPCR microarray analysis as differentially expressed in HCMV-infected fibroblasts compared to uninfected cells (Figure 5).

Among the most altered factors, Caspase 4 and 9 (CASP4, CASP9) resulted upregulated (19.09 and 108.10 fold) at days 4 and 7 p.i., respectively; the same trend was observed for the expression of TNF Superfamily Member 10 (TNFSF10; 6.24 fold) at day 4 p.i.; TNF Receptor Superfamily Member 25 (TNFRSF25) expression was significantly increased at days 0, 7, and 10 p.i. (9.02, 16.48, and 24.29 fold, respectively); Receptor-interacting protein kinase 2 (RIPK2) was upregulated at all infection time points (30.88, 16.79, 6.64, 10.53, and 15.66 fold at 0, 4, 7, 10, and 14 days p.i., respectively). The over-expression of CD27, Cytochrome C (CYCS), Direct IAP-Binding Protein with Low PI (DIABLO), and Tumor Protein 73 (TP73) was observed at day 7 p.i. (14.42, 9.61, 9.11, and 8.17 fold, respectively) and at day 10 p.i. (7.06, 4.84, 4.64, and 5.59, respectively. Additionally, at day 7 p.i., the expression of Myeloid Cell Leukemia 1 (MCL1; 7.35 fold), Nuclear Factor Kappa B Subunit 1 (NFKB1; 5.96 fold), Cell Death Inducing DFFA Like Effector B (CIDEB; 5.15 fold), and TNFRSF1A (5.21 fold) was significantly increased, as was BCL2 Like 1 (BCL2L1; 7.59 fold) at day 14 p.i. and IL-10 at days 7, 10, and 14 p.i. (5.88, 10.21, and 5.68 fold, respectively). BH3 Interacting Domain Death Agonist (BID) was the most upregulated transcript at day 10 p.i. (86.10 fold) and at day 14 p.i (90.51 fold), followed by Tumor Protein P53 Binding Protein 2 (TP53BP2; 37.11 fold at day 10 p.i. and 17.08 fold at day 14 p.i.).

In summary, most of the apoptosis-associated factors were modulated by HCMV infection between 7 and 10 days p.i.

**Figure 6:**
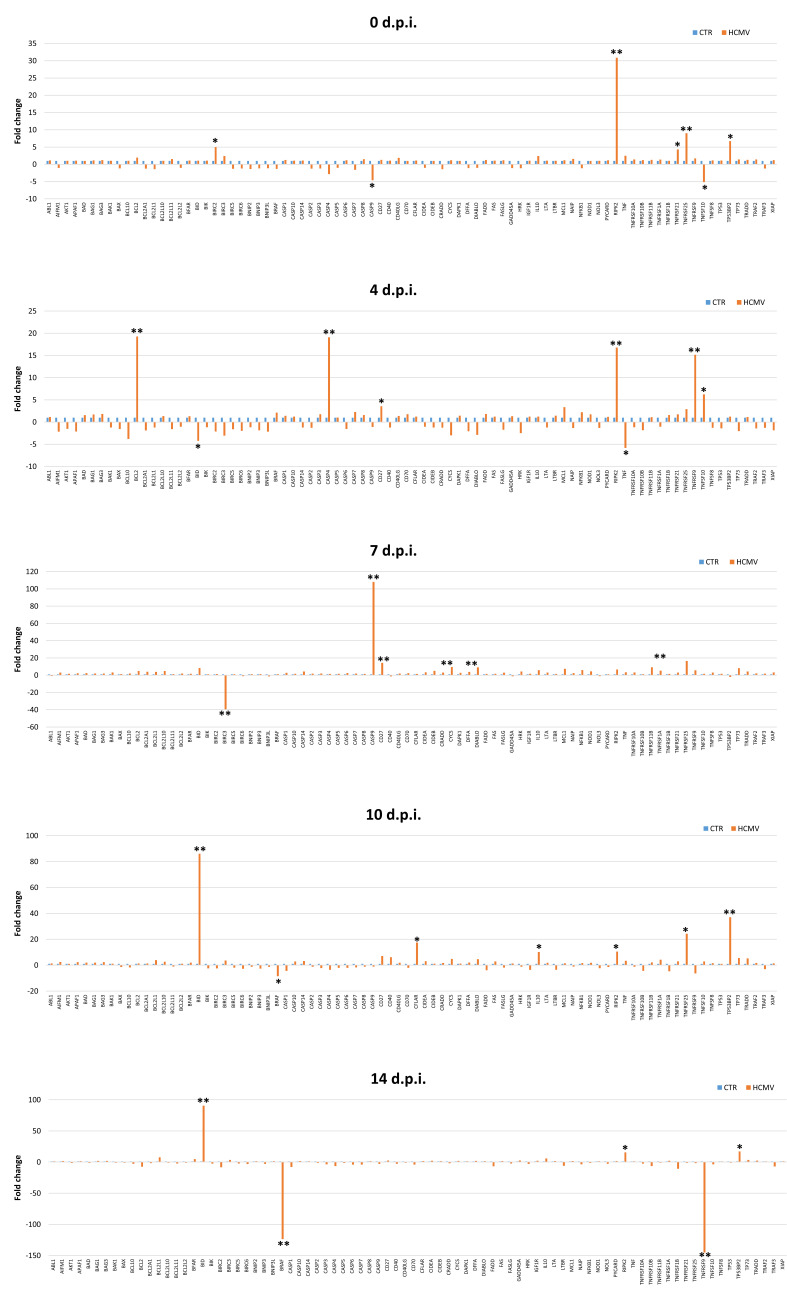
Expression of the apoptosis-associated factors altered by human cytomegalovirus (HCMV) infection in primary human dermal fibroblasts. At each time p.i. (d.p.i. = days post-infection), cell samples were collected and analyzed by specific qPCR microarray. Results are expressed as mean values of fold change (infected cells vs. controls) ± SD of duplicate samples from two independent experiments. * *pc* < 0.01; ** *pc* < 0.001.

On the other hand, while the expression of TNFRSF9 increased at day 4 p.i. (15.17 fold) and at day 7 p.i. (5.73 fold), a significant decrease was observed at day 10 p.i. (−6.43 fold) and at day 14 p.i. (−145.30 fold); also BRAF resulted significantly downregulated at day 10 p.i. (−8.52 fold) and at day 14 p.i. (−123.60 fold), as well as the expression of Baculoviral Iap Repeat-Containing protein 3 (BIRC3) at day 7 p.i. (−39.45 fold). Similarly, at day 14 p.i., several transcripts found upregulated at previous times (e.g., CYCS, DIABLO, TP73, MCL1, NFKB1, CIDEB, TNFRSF1A, TNFSF10, BCL2L1), were no more significantly altered by HCMV infection compared to uninfected control cells or even downregulated, such as CASP4, CASP7, CASP6, CASP3, and CASP9 (−6.45, −4.16, −4.02, −3.64, and −3.9 fold, respectively). A comprehensive summary of the pro-apoptotic factors mostly altered by HCMV infection at the different times p.i. is shown in Figure 6.

**Figure 7:**
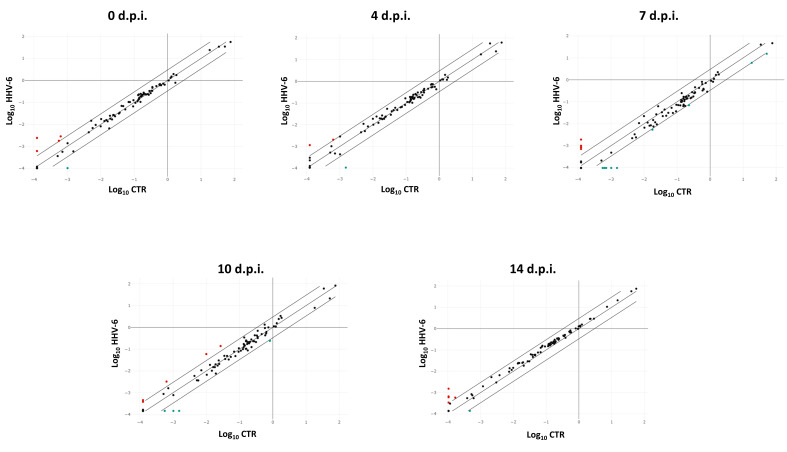
Scatterplot representation of the apoptosis-associated factors altered by human herpesvirus 6A (HHV-6A) infection in primary human dermal fibroblasts. At each time p.i. (d.p.i. = days post-infection), cell samples were collected and analyzed by specific qPCR microarray. The significance threshold was put at 3-fold expression change in infected vs. uninfected control cells. Red dots: upregulated factors; blue dots: downregulated factors; black dots: not significantly altered factors. Results represent mean values of duplicate samples from two independent experiments, and are expressed in logarithmic scale (Log_10_ HHV-6 = logarithmic values in HHV-6A-infected cells; Log_10_ CTR = logarithmic values in control uninfected cells).

By contrast, HHV-6A infection induced a less evident modulation of apoptosis-associated factors in infected cells, compared to HCMV, as displayed in Figure 7.

**Figure 8:**
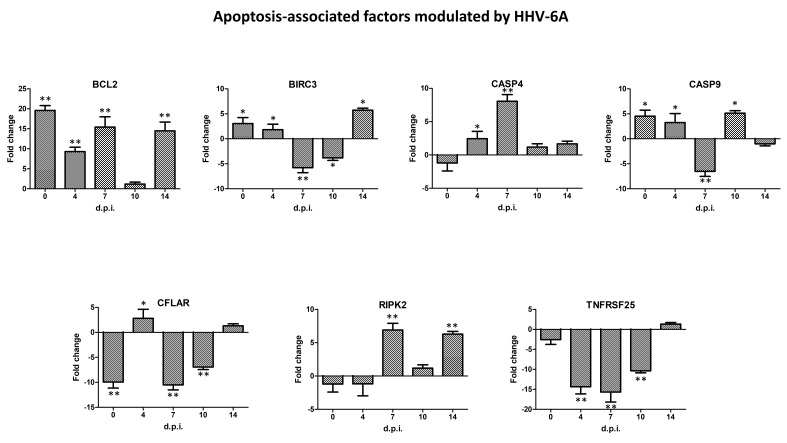
Expression kinetics of apoptosis-associated factors modulated by human herpesvirus 6A (HHV-6A) infection in primary human dermal fibroblasts. At each time p.i. (d.p.i. = days post-infection), cell samples were collected and analyzed by specific qPCR microarray. Results are expressed as mean values of fold change (infected cells vs. controls) ± SD of duplicate samples from two independent experiments. * *pc* < 0.01; ** *pc* < 0.001.

In particular, the only upregulated factor at all times p.i. was B-Cell Lymphoma 2 gene (BCL2; 19.6, 9.31, 15.42, and 14.49, respectively, at 0, 4, 7, and 14 days p.i.). BIRC3 was upregulated at 0 and 14 days p.i. (3.21 and 5.73 fold), whereas it was downregulated at 7 and 10 days p.i. (5.78 and 3.82 fold, respectively). CASP4 was induced only at 7 days p.i. (8.05 fold), while CASP9 was upregulated at 0, 4, and 10 days p.i. (4.52, 3.25, and 5.13 fold) and downregulated at 7 days p.i. (6.53 fold). CFLAR was downmodulated at days 0, 7, and 10 p.i. (9.94, 10.51, and 6.95 fold respectively); RIPK2 resulted induced only at times 7 and 14 days p.i. (6.91 and 6.21 fold). Finally, in contrast to what was observed with HCMV, TNFRSFD25 appeared mostly downregulated by HHV-6A infection (14.34, 15.68, and 10.37 fold respectively at 4, 7, and 10 days p.i.). Figure 8 summarizes the expression kinetics of the HHV-6A induced factors associated with cell apoptosis.

## 3. Discussion

Systemic sclerosis (SSc) is a severe autoimmune disease whose causal agents and pathogenetic mechanisms are still unresolved. The past literature has often associated the infection by human herpesviruses with the onset/development of SSc [19,55,56,57,58], but no definitive data are yet available, especially concerning the possible mechanisms underlying a postulated role of herpesvirus infection in the course of the disease. In particular, beta-herpesviruses HCMV and HHV-6 have been considered possible triggering agents [26,55].

Recent studies have confirmed the high prevalence of beta-herpesvirus infection in SSc subjects, testified by both presence of the viruses at the tissue and/or blood level and by the detection of a significantly higher immune response against HCMV and HHV-6 in SSc patients compared to controls [15,16,26,59]. Furthermore, HHV-6A was shown to be capable of inducing the expression of pro-fibrotic factors in endothelial cells [15], but no information are available about the ability of both HHV-6 and HCMV to induce the expression of fibrosis- and apoptosis-associated factors in human dermal fibroblasts, that are one of the main target cells of the disease.

Since it is widely accepted that the microenvironment plays a significant role in the disease outbreak and progression, the present study aimed to clarify the capacity of such herpesviruses to interfere with the normal metabolism of the infected cells in a way possibly leading to cell fibrosis and/or apoptosis. This aspect was analyzed by in vitro infection assays, using quantitative real-time PCR microarray to detect and quantify simultaneously 84 fibrosis- or apoptosis-associated factors.

Following infection of primary human dermal fibroblasts, both viruses evidenced the ability to induce a potent expression of fibrosis-associated factors, with 22 factors, out of the 84 factors analyzed, altered by the infection of one or both viruses. In detail, HCMV appeared most powerful compared to HHV-6A, inducing a higher number of factors and at a higher extent than HHV-6A.

In particular, HCMV infection led mainly to the over-expression of CCL2, CCL3, CCL11, CXCR4, IL-1β, IL13, MMP1, MMP3, MMP9 and MMP13, SERPINA1, TNFα, and BMP7, and the gradual and constant downregulation of IL13RA2 (up to almost 800-fold at 14 days p.i.).

Additionally, HHV-6A exhibited a profound modulating effect on several fibrosis-associated factors. In particular, CXCR4 resulted highly upregulated at 4 and 7 days p.i. (up to >36 fold), IL-1β was constantly upmodulated (up to >7 fold), four MMPs were overexpressed (MMP1-3-9-13, up to 21 fold), and also IL-4 (>51 fold), IL-10 (>20 fold), and TNFα (>28 fold) were upregulated by the virus, together with BMP7 (>16 fold), similarly to HCMV.

Notably, most of the analyzed pro-fibrotic factors were found to be overexpressed upon infection by HCMV and/or HHV-6A, suggesting that the viral infection (and likely coinfection) might have a significant impact on the cell microenvironment. Considering that virus-infected cells were not selected by cell-sorting or other methods before RNA analysis, and thus could not represent 100% of the analyzed cells, the observed high fold-change values and significance might be even underestimated, further supporting a strong effect of both viruses on fibrosis modulation. In addition, some key inducers of cell fibrosis were significantly upregulated by both viruses, suggesting that in coinfections they may synergize and have an even higher effect on infected cells. Such factors included CXCR4, IL-1β, MMP1, MMP13, and TNFα.

Among them, CXCR4 (upmodulated by both viruses) is overexpressed the skin of SSc patients [60] and is known to contribute to fibrosis [60,61].

IL-1β is induced upon toll-like receptor activation and exerts pro-fibrotic effects by inducing other pro-inflammatory cytokines, release of fibrosis markers, TGF-β synthesis, and fibroblasts proliferation [62,63,64]. To this regard, recent findings show that the expression of most IL-1 family cytokines, such as IL-1β, are abnormal in many autoimmune diseases including SSc. In patients with SSc, there is an increase of IL-1β in the serum and bronchoalveolar lavage fluid [65]; furthermore, in the lesion skin tissue of SSc patients, the expression levels of IL-1β are significantly upregulated and there is a positive association between dermal fibrosis severity evaluated by modified Rodnan skin score (mRSS) and IL-1β expression [66].

A dysregulated expression of MMPs has been observed in subjects with pulmonary fibrosis [67], where they have been shown to have a profound impact on the mechanisms involved in fibrosis development. In particular, MMP1 overexpression has been associated to the pathogenesis of fibrosis, MMP3 is induced in epithelial to mesenchymal transition, MMP9 is induced by TGF-β and has a pro-fibrotic action, and MMP13 has a controversial pro- and anti-fibrotic action on fibroblasts [68].

TNFα has a well-recognized role in the induction of SSc and TNFα blockage has anti-fibrotic therapeutic effect; intriguingly the TNF superfamily member lymphocyte T-related inducible ligand LIGHT, competing for gD binding for herpesvirus entry on T cells, is overexpressed in SSc [69].

Another factor induced by both viruses is BMP7, which however possesses a prevalently recognized anti-fibrotic role, although somehow controversial [70,71].

Besides, other factors affected individually by the two viruses are of note. IL-13 (upregulated by HCMV > 21 fold) has been shown to play a role in many inflammatory and fibrotic diseases, including SSc, and appears to be necessary in the effector phase of inflammation and fibrosis [72,73]. In particular, IL-13 is significantly expressed in skin biopsies of SSc patients and its levels were found markedly increased in parallel with the progression of cutaneous fibrosis in bleomycin-induced SSc murine model [74,75]. CCL2, CCL11, and SERPINA1 transcripts were also upregulated by HCMV (up to 39, 43, and 22 fold, respectively). CCL2 was strongly expressed in skin biopsy samples from patients with early dcSSc and a number of studies have confirmed the upregulation of both protein and mRNA in SSc [76,77]; its levels were found higher in dSSc and they correlated with mRSS [78]. CCL11 was found to be significantly altered in the serum of preclinical/early SSc patients [79]. SERPINA1 gene expression has been reported as associated to pulmonary fibrosis and regulation of immune response [80].

CCL3 (upregulated by HCMV > 48 fold) has been demonstrated to play a role in dermal and pulmonary fibrosis in a murine sclerodermatous disease model [81] and, recently, CCL3 transcripts were found increased in skin biopsies of SSc patients [74].

Intriguingly, overexpression of IL-13 receptor alpha 2 (IL13RA2) protects against fibrosis [82]; instead, it was strongly downmodulated by HCMV, up to almost 800 fold.

IL-4 (induced by HHV-6A, >51 fold) is a recognized pro-fibrotic factor, identified since over 20 years as a critical cytokine, increased in the blood, bronco-alveolar lavage cells and skin of SSc patients [83]. It is also known that IL-4 is a potent activator, more active than TGF-β, in inducing collagen synthesis in human skin fibroblasts [84,85], and can drive fibroblast differentiation and promote pro-fibrotic macrophages activation [86]. Interestingly, the IL-4/IL-13 axis exerts a key role in skin fibrosis and scarring [87], again suggesting a possible cooperation of the two herpesviruses in fibrosis induction. Furthermore, HHV-6A specifically induced ITGB6, whose expression is restricted to epithelial cells and associated with fibrosis [88].

The pattern of pro-fibrotic factors induced by HHV-6A in fibroblasts was similar but not completely superimposable with that observed in endothelial cells, where IL-4, TNFα, and MMP9 were similarly induced [15], but IL-5 was also increased, in contrast to what was detected in fibroblasts, suggesting that viral strategies depend, at least partly, on the cell type and microenvironment.

By summarizing the results obtained in this in vitro study on the modulation of several pro-fibrotic factors induced by HCMV and/or HHV-6A, it is worthy to note that although a number of them are often altered in different autoimmune diseases [89], most of them have been described to be up- or downregulated in SSc, as already highlighted above [15,60,61,62,63,64,65,66,67,68,69,70,71,72,73,74,75,76,77,78,79,80,81,82,83,84,85,86,87,88].

Further factors described in the literature, such as CXCL10, IL8, and IL6, not included in the panel analyzed here, might deserve future investigation, as also involved in SSc onset and fibrosis progression [90,91,92,93,94].

Differently from fibrosis-associated factors, the impact on apoptosis-related factors was quite different for the two viruses, with a clear potent and significant upregulation effect caused by HCMV, less evident for HHV-6A. Namely, HCMV infection altered significantly the expression of 20 factors out of the total 84 factors tested, whereas HHV-6A affected significantly only seven factors.

Among the most HCMV-altered factors were CASP4, CASP9, TNFRSF25, RIPK2, BID, TP53BP2, CD27. Other HCMV-modulated factors, although to a lesser extent, were CYCS, DIABLO, TP73, MCL1, NFKB1, CIDEB, TNFRSF1A, TNFSF10, BCL2L1, IL-10, BIRC3; notably, all these transcripts were no longer significantly altered by HCMV infection compared to uninfected control cells at day 14 p.i.; moreover, at this time p.i. CASP4, CASP7, CASP6, CASP3, and CASP9 resulted downregulated.

As to HHV-6A, only BCL2 was constantly upregulated (>19 fold), whereas the other factors exhibited a bi-phasic trend, with upregulation at certain times p.i. and downregulation at the others. Altered factors included BIRC3, CASP4, CASP9, CFLAR, RIPK2, and TNFRSF25 (mostly downregulated by the virus).

The observed more effective HCMV capacity of modulating pro-fibrotic and pro-apoptotic factors compared to HHV-6A could be connected, for instance, to the recent ex vivo investigations from our group showing significant correlations between HCMV-specific T cell responses in SSc patients and clinical parameters of worse disease outcome (longer duration and higher values of mRSS) [16].

To date, the involvement of apoptosis in the development of dermal sclerosis is unclear. As apoptosis plays an important role in the normal resolution process, its alteration may lead to pathologic conditions. In a murine model of bleomycin-induced fibrosis, it has been speculated that at sufficiently high levels of apoptosis, the skin clearance system may be impaired by the need to remove apoptotic cells. This interference on the resolution of the inflammation may lead to secondary necrosis of apoptotic cells, inducing skin tissue damage and a fibroproliferative response [95].

Overall, several factors are shared by the two viruses, suggesting a common pattern of action, as expected being both beta-herpesviruses with common tropism and pathogenetic characteristics. However, some actions are peculiar of HCMV or HHV-6A, indicating that they may have also different actions, likely potentiating each other, as already known for other diseases [96,97,98,99,100].

It is known that HCMV and HHV-6 can interact by reactivating each other, thus one virus may potentiate the effect of the other virus in co-infected patients [96,97,98,99,100]. Since both HCMV and HHV-6 are highly prevalent in the human population, the coinfection is a very probable event in one subject. Thus, it might be hypothesized that in subjects with impaired ability to control herpesvirus infection/reactivation, the simultaneous presence of both viruses might lead to even worse effects compared to those resulting from a single infection. This aspect should deserve future investigation, including simultaneous infection by the two viruses, using also different types of cells and suboptimal virus concentration, in order to observe a postulated effect of virus cooperation with likely potentiating effects.

Although establishing a causal role in complex diseases such as SSc is always very difficult, especially for widespread viruses such as HCMV and HHV-6, the data collected here suggest that both viral agents might have a relevant role in the induction of cell fibrosis at the tissue level, and open new perspectives about the potential therapeutic use of anti-herpetic drugs able to block the progression of SSc, especially in the very early stages of the disease.

## 4. Materials and Methods

### 4.1. Cell Culture

Primary human dermal fibroblasts (adult skin) (Lonza, Basel, Switzerland) were seeded in 25 cm^2^ flasks and maintained in complete fibroblast cell medium (Fibroblast Cell Basal Medium), supplemented with 2% fetal bovine serum, 0.1% r-human fibroblast growth factor-B, 0.1% insulin, 0.1% gentamicin sulphate/amphotericin-B (“Bullet Kit”) (Lonza, Basel, Switzerland). Sub-cultivation was performed at approximately 80% confluence using the “ReagentPack Subculture Reagent Kit” (Lonza, Basel, Switzerland), according to the manufacturer’s instructions.

### 4.2. Virus Strains and Titration

HCMV TB40E reference strain (kindly provided by Prof. Thomas Mertens, Institute of Virology, Ulm University, Ulm, Germany) was propagated in MRC5 cells; the viral infectious titer was determined as previously described [101]. The same stock was used for all the infections (viral titer: 10^9^ PFU/mL).

HHV-6A (U1102 strain) was obtained in J-Jhan T cells as previously described and contained about 1010 genome equivalents per mL. The same stock was used for all the infections [102].

### 4.3. Viral Infection

Primary human dermal fibroblasts at 90% confluence were infected with the TB40E strain of HCMV at a multiplicity of infection (MOI) of 0.1, or with the U1102 strain of HHV-6A at a MOI of 1.0. The infected cells were incubated at 37 °C for 2 h. At the end of the adsorption period, the virus inoculum was removed and replaced with complete fibroblast cell medium. Cells were incubated at 37 °C for 0, 4, 7, 10, and 14 days. At the indicated times, cells were collected by scraping, washed in cold PBS, and pelleted by centrifugation for 10 min at 1000× *g*. Cell pellets were instantaneously frozen in liquid nitrogen and kept at −80 °C until use. Two aliquots per sample were prepared, for respective extraction of total DNA and RNA.

### 4.4. DNA Extraction and Quantitative Real-Time PCR (qPCR) Assay

Total DNA was extracted from infected cells using the NucliSENS^®^ EasyMAG^®^ platform (bioMérieux, Marcy-l’Étoile, Francia). The DNA was subjected to qPCR amplification using the CMV ELITe MGB^®^ Kit (ELITechGroup, Turin, Italy) for the detection and quantification of the human HCMV DNA exon 4 region of the immediate-early (IE)1 gene. The assay was performed according to the manufacturer’s instructions using the 7500 Real-time PCR system (ABI PRISM, Applied BioSystems, Foster City, Canada, USA). The results were expressed as DNA copies/mL (logarithmic scale).

For HHV-6A quantification, a specific qPCR amplifying the U94 viral gene was used, as previously described [103]. The assay was performed in a 7500 Real-time PCR system (ABI PRISM, Applied BioSystems, Foster City, Canada, USA) and the results were expressed as DNA genome copy number per µg of total DNA, corresponding to about 10^5^ cells.

### 4.5. RNA Extraction

Total RNA was extracted from infected and uninfected cell pellets by the mirVana™ PARIS™ RNA and Native Protein Purification kit, following the manufacturer’s instructions (Invitrogen, Thermo Fisher Scientific, Milan, Italy). Extracted RNA was checked and quantified by spectrophotometric reading at 260 and 280 nm wavelength, using a Nanodrop. Elimination of contaminant DNA was assured by DNase I digestions (Thermo Fisher Scientific, Milan, Italy) and absence of contaminating DNA was assessed by amplifying an aliquot of extracted RNA for human β-actin gene. After verifying that the samples were devoid of contaminating DNA, 1 µg aliquots of total RNA were retrotranscribed by RT2 First Strand kit (Qiagen, Hilden, Germany) according to manufacturer’s instructions. Briefly, RNA template was mixed with reverse-transcription master mix and incubated for 15 min at 42 °C, and then 5 min at 95 °C for enzyme inactivation. Following retrotranscription, 500 ng of cDNA were used for subsequent analysis by qPCR microarray.

### 4.6. qPCR Microarray Analyses

The expression of factors associated with fibrosis or apoptosis in infected cells was analyzed by qPCR microarray. In detail, two different microarrays were used, one targeted to fibrosis-associated factors and the other to apoptosis-associated factors (both by Qiagen, Hilden, Germany), both simultaneously detecting and quantifying 84 cellular factors respectively associated with fibrosis or apoptosis. Results represent up- or down-modulation of each factor in infected vs. uninfected control cells, and are expressed as fold-change values compared to control values after normalizing for six housekeeping genes (βactin, β2microglubulin, GAPDH, HPRT1, RPLP0, and HGDC), as calculated by the specific Qiagen software (https://geneglobe.qiagen.com/ca/analyze/). Reported results are expressed as mean fold value ± SD of duplicate samples from two independent experiments. Analysis threshold was put at 3-fold change of up- or down-modulation.

### 4.7. Statistical Analyses

Student’s *t*-test was used for statistical analyses (*p* ≤ 0.05 was considered significant). For multiple comparisons (microarray data), the Bonferroni correction was applied, and a corrected *p* value (*pc*) ≤ 0.05 was considered significant.

## Figures and Tables

**Table 1:** Human cytomegalovirus (HCMV) DNA quantitation in infected primary human dermal fibroblasts at the indicated times post-infection (p.i.).

Times of Infection	DNA Copies/mL *		DNA Copies/mL (log_10_)		Standard Deviation
0 days	-	-	-	-	-
4 days	1.11 × 10^5^	1.16 × 10^5^	5.057	5.063	0.004
7 days	2.93 × 10^5^	2.97 × 10^5^	5.466	5.473	0.005
10 days	5.95 × 10^5^	6.01 × 10^5^	5.775	5.780	0.003
14 days	1.36 × 10^7^	1.38 × 10^7^	7.135	7.141	0.004

* Mean values of HCMV genome copy number per mL with related standard deviations (SD) from two independent experiments are shown.

**Table 2:** Human herpesvirus 6A (HHV-6A) DNA quantitation in infected primary human dermal fibroblasts at the indicated times p.i.

Times p.i.	DNA Copies/μg *		DNA Copies/µg (log_10_)		Standard Deviation
0 days	-	-	-	-	-
4 days	5.56 × 10^5^	5.68 × 10^5^	5.745	5.754	0.006
7 days	9.85 × 10^5^	9.72 × 10^5^	5.993	5.987	0.004
10 days	5.63 × 10^5^	5.65 × 10^5^	5.750	5.752	0.001
14 days	8.74 × 10^4^	8.90 × 10^4^	4.941	4.949	0.006

* Results are expressed as mean values of genome copy number per µg of total DNA (corresponding to about 10^5^ cells) from two independent experiments ± SD.
